# One-Stage Endovascular-to-Open Repair for Traumatic Common Carotid Artery (CCA) Pseudoaneurysm: Expanding the Hybrid Treatment Landscape

**DOI:** 10.7759/cureus.86155

**Published:** 2025-06-16

**Authors:** Alexander T Daskalov, Boris Ilchev

**Affiliations:** 1 Vascular Surgery, Acıbadem City Clinic Tokuda Hospital, Sofia, BGR

**Keywords:** common carotid artery, endovascular stenting, hybrid repair, pseudoaneurysm, trauma surgery

## Abstract

We report the case of a 62-year-old male who presented with a gradually enlarging, pulsatile cervical mass four months after sustaining a penetrating neck injury. Imaging revealed a 28 × 20 mm pseudoaneurysm of the left common carotid artery (CCA), located 15 mm proximal to the bifurcation, without evidence of active bleeding. Given the lesion’s size, delayed presentation, and proximity to the carotid bifurcation, a single-stage hybrid treatment strategy was pursued. An endovascular covered stent was first deployed to achieve immediate haemodynamic stabilisation and maintain cerebral perfusion. This enabled safe and controlled surgical dissection during the same operative session. Definitive open repair was then performed, including resection of the pseudoaneurysm and interposition of a reversed autologous femoral vein graft between the CCA and the carotid bifurcation. Postoperative imaging confirmed graft patency, and the patient experienced no new neurological deficits. To our knowledge, this represents the first reported case of a traumatic CCA pseudoaneurysm in an adult managed with a single-stage approach in which a temporary endovascular stent was used as a bridge to definitive open reconstruction. This novel strategy highlights the expanding role of hybrid techniques in vascular trauma and underscores the value of surgical versatility in the management of complex arterial injuries.

## Introduction

Traumatic pseudoaneurysms of the extracranial carotid arteries are rare, accounting for less than 1% of all arterial aneurysms, with those involving the common carotid artery (CCA) being particularly uncommon [1]. These lesions, often resulting from penetrating trauma, lack a true arterial wall, placing them at high risk for rupture or thromboembolic complications [2].

Management remains challenging due to the rarity and variable presentation of these injuries. While open surgical repair has long been the standard, it is associated with notable morbidity, including cranial nerve injury [3]. Endovascular techniques, particularly the use of covered stents, have emerged as a less invasive and increasingly preferred option for select patients [4].

In complex cases, such as large defects, involvement of branch vessels, or uncertainty about active bleeding, a hybrid approach combining endovascular and open surgical strategies may offer distinct advantages [5]. These techniques allow for temporary bleeding control and cerebral perfusion via stenting, followed by definitive open reconstruction, thus leveraging the strengths of both modalities.

To the best of our knowledge, this is the first documented case in which a covered stent was employed as a temporary bridge to facilitate subsequent open repair in a traumatic carotid artery injury.

## Case presentation

History

We report the case of a 62-year-old male who presented with a gradually enlarging, pulsatile mass in the left cervical region, four months after sustaining a stab wound to the left side of the neck. The initial traumatic event occurred during a physical altercation, following which the patient was urgently transferred to an outside facility in hemorrhagic shock. His course was complicated by an ischemic stroke with resultant right-sided hemiparesis and aphasia. No documentation from the initial hospitalization was available upon presentation. Notably, there was no visible surgical scar in the neck region, suggesting that no definitive vascular repair had been performed at the time. The patient did not receive definitive vascular repair during the initial hospitalization and was lost to follow-up until presenting four months later with a progressively enlarging neck mass.

Examination and imaging

**Figure 1:**
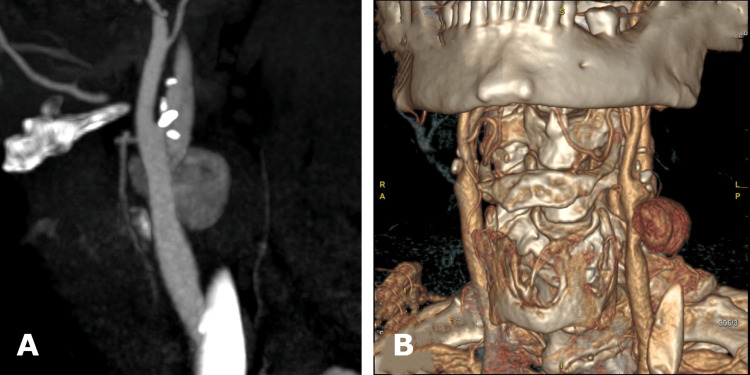
CTA revealing a 28 × 20 mm pseudoaneurysm of the left common carotid artery (CCA), located approximately 15 mm proximal to the carotid bifurcation, without evidence of active extravasation. A) 2D sagittal image of the pseudoaneurysm; B) 3D reconstruction of the pseudoaneurysm.

On examination, the patient reported a progressively enlarging cervical mass. CTA revealed a 28 × 20 mm type V pseudoaneurysm of the left CCA, located approximately 15 mm proximal to the carotid bifurcation, without evidence of active bleeding (Figure 1).

Additional findings included thrombosis of the right internal jugular vein. His past medical history was significant for bipolar disorder, which was under pharmacological treatment.

Surgical management

Given the pseudoaneurysm’s size, proximity to the bifurcation, and risk of rupture, single-stage surgical intervention was performed. Through a small longitudinal cervical incision, we accessed the proximal CCA and introduced a 10 Fr vascular sheath. A 10 × 27 mm Bentley covered stent was deployed across the CCA and internal carotid artery (ICA) to secure the lesion and serve as a temporary conduit, effectively eliminating the need for initial arterial clamping and allowing controlled dissection of the surrounding structures. The stent also functioned as a shunt, maintaining cerebral perfusion during the dissection. Although the stent initially controlled the lesion and maintained perfusion, it was later removed to allow for definitive anatomic reconstruction with a more durable conduit.

**Figure 2:**
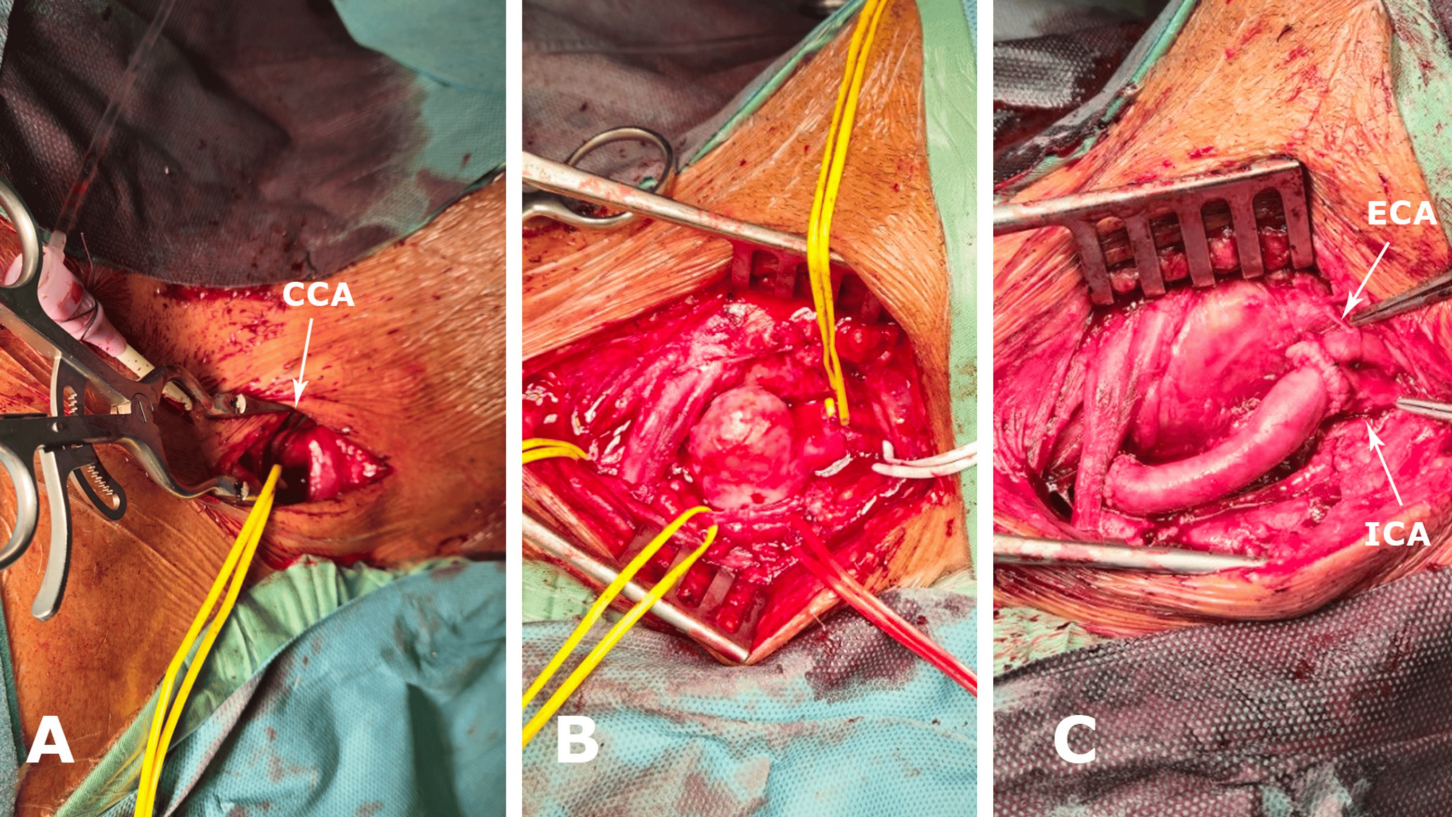
Major stages of the procedure with the final result. A) Small longitudinal incision of the common carotid artery (CCA) and insertion of a 10 Fr sheath in an antegrade fashion to facilitate stent implantation. B) Dissection of the aneurysm and carotid bifurcation. C) Construction of the interposition graft between the CCA and the carotid bifurcation (internal carotid artery (ICA) and external carotid artery (ECA)).

Due to the size of the arterial defect, primary repair was not feasible. Therefore, a 7-cm interposition graft was fashioned using the femoral vein harvested distal to the deep femoral vein. Systemic heparinisation with 5000 IU of heparin was initiated. After a short period (less than two minutes) of cross-clamping, the pseudoaneurysm was incised using a longitudinal incision, and the covered stent was explanted. A three-lumen Pruitt-Inahara shunt was then inserted to preserve cerebral perfusion. Following complete resection of the diseased arterial segment, the graft was reversed and sutured between the CCA and the carotid bifurcation, performing an end-to-end anastomosis proximally and distally (Figure 2).

Postoperative course

**Figure 3:**
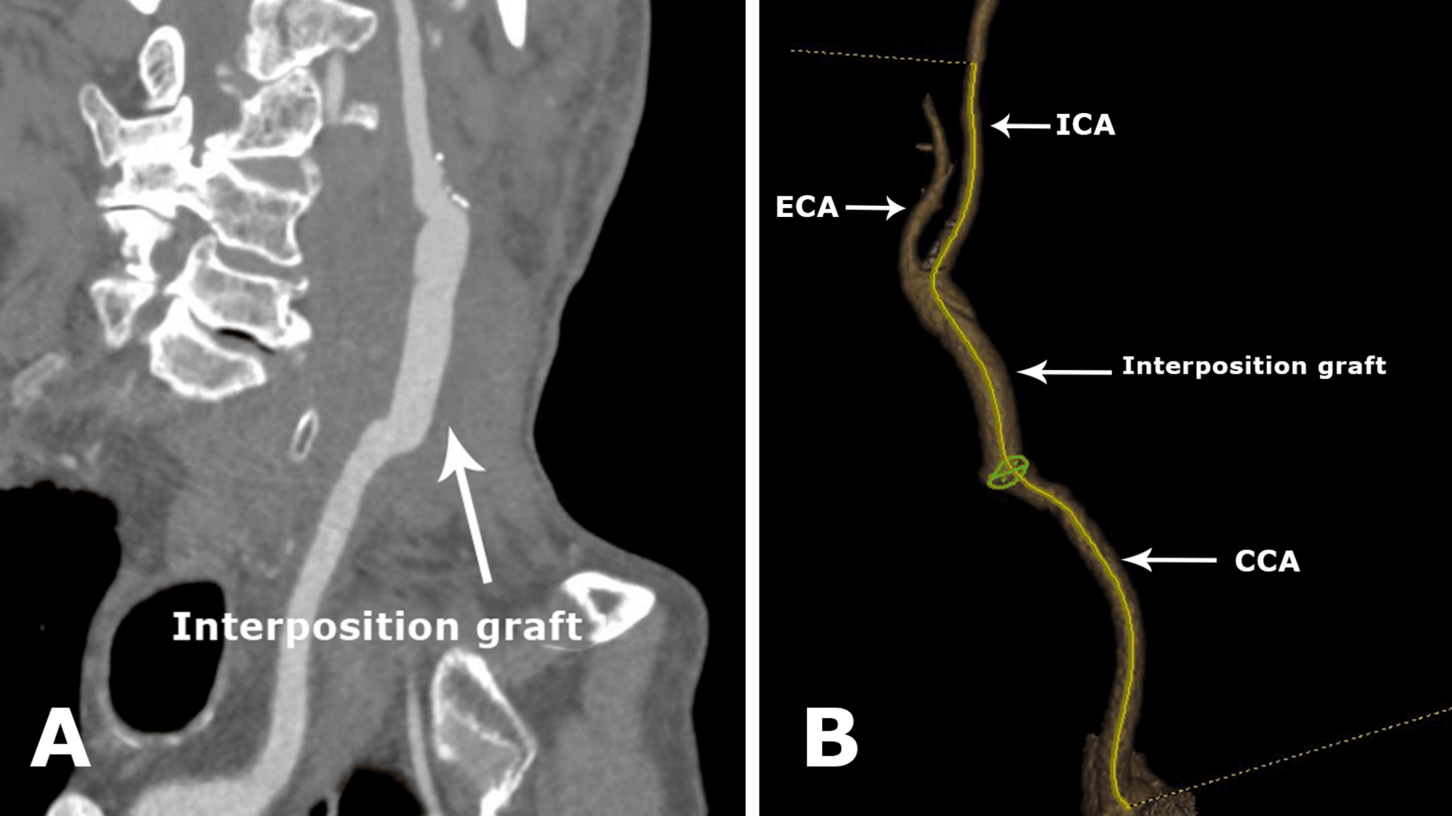
Postoperative computed tomography angiogram (CTA) showing patent reconstruction. A) 2D reconstruction of the interposition graft. B) 3D reconstruction showing a patent common carotid artery (CCA), interposition graft, external carotid artery (ECA), and internal carotid artery (ICA).

Postoperatively, the patient remained neurologically intact, with no new deficits. CTA performed on postoperative day one confirmed patency of the reconstruction and appropriate positioning of the graft (Figure 3). The patient did not experience any complications related to the deep vein harvesting.

He was discharged on postoperative day eight on dual antithrombotic therapy: rivaroxaban 5 mg twice daily and aspirin 100 mg once daily. Follow-up was conducted at 3 and 6 months using Doppler ultrasound. The reconstruction remained patent with no signs of stenosis. Long-term surveillance will be essential to monitor graft patency and prevent late complications.

## Discussion

Traumatic pseudoaneurysms of the CCA are rare but potentially lethal injuries. They account for approximately 1% of trauma cases [6], yet can lead to catastrophic outcomes if left untreated. Pseudoaneurysm formation represents arterial wall injury contained by surrounding tissue and thus carries high risks of rupture and thromboembolic complications. Patients may present with a pulsatile neck mass, neurologic deficits due to nerve compression, or signs of embolization. Given the danger of sudden rupture with fatal hemorrhage or stroke, prompt definitive treatment of CCA pseudoaneurysms is imperative [7].

In adults, these lesions most often result from penetrating or blunt trauma, as opposed to true aneurysms, which are exceedingly uncommon in the carotid circulation [7]. The need for rapid control of hemorrhage while preserving cerebral perfusion makes the management of carotid pseudoaneurysms particularly challenging and significant in the trauma setting.

Historically, open surgical repair was the gold standard therapy for extracranial carotid pseudoaneurysms. Standard open techniques include proximal and distal control of the artery with resection of the pseudoaneurysm, followed by either ligation or reconstruction (e.g., primary anastomosis or interposition graft) [7]. This approach effectively removes the diseased segment and can utilize autologous grafts, eliminating the need for foreign material. Open repair remains preferred in certain scenarios (such as accessible zone II neck injuries) due to the ability to address associated aerodigestive injuries and perform definitive vascular reconstruction in one setting [8].

However, open surgery in the neck carries significant risks and technical challenges. Exposure of the carotid artery in hostile or deep locations (zone I at the thoracic inlet or high zone III near the skull base) can be difficult and hazardous [8]. Even in experienced hands, open surgical management of carotid injuries has reported perioperative stroke rates approaching 9%, along with notable mortality [6]. Additionally, surgical dissection in the neck risks cranial nerve injury, reported in approximately 6-9% of carotid surgeries [9]. These morbidity rates underscore the limitations of open repair in complex carotid trauma.

Over the past two decades, endovascular stent grafting has emerged as an effective, less invasive alternative for carotid pseudoaneurysms. Covered stent grafts can be deployed via an endovascular approach to exclude the pseudoaneurysm from within, avoiding a large neck incision [10].

Published case series demonstrate high technical success with endovascular repair, and this approach is associated with lower immediate morbidity compared to open surgery [7].

A covered stent provides a physical barrier that seals the arterial defect while maintaining lumen patency, thus achieving immediate hemorrhage control and preserving blood flow [6]. This is particularly advantageous in anatomically challenging zones where surgical clamping would be difficult [8]. Indeed, endovascular treatment has been shown to overcome many anatomic limitations of open surgery and, in some reports, has yielded lower early mortality in trauma patients [6].

Each modality has distinct benefits and drawbacks in the adult trauma patient:

Open repair

Open repair allows direct visual control of the artery and use of autologous grafts (e.g., saphenous or femoral vein (FV)), which are resistant to infection and durable in the long term [11]. Open surgery definitively removes the damaged segment. However, it requires time-consuming dissection and carotid cross-clamping, which can precipitate cerebral ischemia. There is also a risk of stroke during arterial clamping or reperfusion [12], as well as the aforementioned cranial nerve injuries and surgical wound complications. Open repair may be contraindicated or very high-risk in unstable patients or anatomically inaccessible injuries.

Endovascular stent-graft

Endovascular stent-graft avoids a major operation and can be performed rapidly, even in unstable patients. A covered stent graft achieves immediate exclusion of the pseudoaneurysm while maintaining continuous cerebral perfusion [6]. This minimally invasive approach avoids the morbidity of a neck incision and cranial nerve trauma. Endovascular repair is particularly useful for high or low cervical lesions (zones III and I), where surgical exposure would necessitate a mandibular dislocation or sternotomy [8]. The main drawbacks of stent-grafting are the need for antithrombotic therapy and concerns about long-term durability. Patients require dual antiplatelet therapy around the procedure and prolonged aspirin use to mitigate the risk of stent thrombosis or in-stent stenosis [6]. In the acute trauma setting, the required antiplatelet medication can complicate management of concomitant injuries (e.g., traumatic brain injury or solid organ injury with bleeding risk). Furthermore, placing a foreign stent in potentially contaminated wounds raises infection risk, and stent grafts are not ideal in cases of infected pseudoaneurysms. Lastly, endovascular skill and resources must be immediately [8] available, and device placement itself carries a risk of distal embolization or occlusion if not carefully managed [8].

A hybrid strategy, combining endovascular and open techniques, was employed in this case to capitalize on the advantages of both approaches [13]. The patient’s CCA pseudoaneurysm was first managed with an emergent endovascular covered stent, which immediately tamponaded the bleeding and preserved carotid perfusion. Subsequently, an open surgical repair was carried out, during which the injured arterial segment (along with the stent and pseudoaneurysm) was resected and replaced with an autogenous FV graft. This two-staged approach is innovative for traumatic carotid pseudoaneurysms. To our knowledge, there are no prior reported cases in the literature of a traumatic CCA pseudoaneurysm in an adult treated with a covered stent as a temporary bridge to definitive open reconstruction. This case underscores the unique versatility of the vascular surgeon, who is trained to perform both open and endovascular procedures and, when appropriate, integrate them into a hybrid approach. This dual skill set enables comprehensive management of complex vascular injuries, including the complete resection of pseudoaneurysms. Such an approach not only ensures definitive treatment but also eliminates the risk of leaving behind a residual cervical mass, which could lead to delayed complications such as cranial nerve compression, an outcome less readily addressed by specialties focused solely on endovascular therapy.

Previous reports in adults have typically utilized either open repair or endovascular stenting alone [7]. The concept of using a covered stent graft as a temporary measure has been described mainly in the context of infected or malignant carotid pseudoaneurysms (so-called “carotid blowout” syndrome). In those cases, a stent can control hemorrhage to allow stabilization or infection control before surgery [8].

However, applying a one-stage endovascular-then-open approach in a trauma setting is novel. This hybrid technique allowed us to achieve immediate bleeding control and neuroprotection (via the stent), giving us time to carefully dissect the defect without the need for clamping. The open approach, by contrast, does not offer clear predictability regarding when the brain will be reperfused, especially in scenarios where the surgeon must first gain control of active bleeding before considering cerebral perfusion. By subsequently performing an open interposition graft, we provided a durable biologic reconstruction more suitable for our patient once the acute danger had passed. The success of this approach in our case suggests that a covered stent can serve as an effective bridge to surgery in select adult carotid trauma-though it has not been previously reported for CCA pseudoaneurysms in this population. This novel strategy fills a management gap: it offers an option when neither open nor endovascular treatment alone is optimal, essentially combining their strengths (rapid hemorrhage control and definitive long-term repair).

Maintenance of cerebral perfusion during carotid artery injury repair is a paramount concern, as even short intervals of interrupted flow can cause ischemic brain injury. In open surgery for carotid trauma, surgeons often employ temporary intraluminal shunts to ensure continuous cerebral blood flow while repairing the vessel. For example, in a recent report of open repair of a penetrating CCA injury, a Pruitt-Inahara shunt was inserted to restore flow prior to graft interposition [12].

In that case, the shunt likely shortened the brain ischemia time, although the patient still suffered a transient cerebral infarction attributed to ischemia-reperfusion injury [12]. This underscores that even with careful technique, the period of clamping and repair carries a risk of neurologic deficit. Neuroprotective measures such as shunting, selective brain perfusion, or expedited anastomosis are therefore critical considerations in open carotid repairs.

The endovascular-first stage of our hybrid approach functioned as an immediate intravascular shunt while also definitively sealing the arterial injury. By deploying a covered stent, we avoided any prolonged carotid cross-clamping altogether in the initial emergent phase, continuous antegrade perfusion to the brain was preserved throughout. This likely contributed to the absence of neurologic deficit in our patient. The stent graft excluded the pseudoaneurysm, preventing further hemorrhage or distal embolization of thrombus, which is another mode of neuroprotection (reducing the risk of stroke from embolic debris). Importantly, because the stent was in place, the subsequent open surgery could be performed in a more controlled fashion with the pseudoaneurysm already secured. Some authors have also described using distal embolic protection devices or performing a flush of the ICA after stent deployment to wash out any debris before finalizing the repair [14], though in an emergency trauma context such measures must be balanced against time. In our case, the initial stenting obviated the need for urgent shunt placement or hurried anastomosis, thereby simplifying cerebral protection.

Avoiding hypotension, minimizing carotid clamp duration, and ensuring adequate collateral circulation (via the circle of Willis or a shunt/stent) are all essential to prevent stroke during such interventions [12].

In our patient, the absence of any neurologic sequelae suggests that cerebral perfusion was effectively protected by the chosen management sequence.

The long-term considerations in carotid artery injury repair include the patency of the reconstruction, risk of restenosis, and potential need for reintervention over time. Each repair modality carries distinct implications in this regard. Endovascular stents in the carotid position require ongoing antiplatelet therapy (typically lifelong aspirin, with an initial period of dual therapy) to mitigate thrombosis [6].

Even with medical therapy, in-stent restenosis due to intimal hyperplasia is a known issue after carotid stenting. Trauma-specific series have reported notable occlusion rates for carotid stent grafts on follow-up, for example, a literature review by Maras et al. noted about a 15% stent occlusion rate in traumatic extracranial ICA pseudoaneurysms [8]. In a series of blunt carotid injuries, Cothren and colleagues observed a 45% occlusion rate in patients treated with stents, compared to a 5% occlusion rate with medical management alone [8]. While covered stents can be life-saving in the acute setting, these data indicate that their long-term durability in trauma patients may be inferior, potentially due to the high-flow environment and endothelial injury prompting neointimal proliferation. Additionally, late complications of indwelling stents such as fracture, infection, or erosion into adjacent structures have been reported in carotid applications (particularly in the context of infection or radiation) [8]. Because of these concerns, purely endovascular treatment of carotid pseudoaneurysms raises questions about future restenosis and the need for surveillance and possible reinterventions (e.g., repeat angioplasty or stent revision).

In contrast, open surgical reconstruction with an autogenous graft offers excellent long-term patency in carotid artery repairs. Autologous vein grafts, such as the FV graft used in our case, have the advantages of biocompatibility and durability. FV grafts are of appropriate caliber for the carotid artery and have robust walls, making them resistant to dilation and rupture [11]. In a series of 10 patients who underwent carotid resection and reconstruction with FV grafts (for tumor involvement of the carotid), graft patency was 100% with no thromboses at two-year follow-up [11]. While trauma patients differ from elective surgical patients, this experience supports the expectation of excellent mid-term graft durability. Autogenous grafts are also less prone to infection than synthetic materials or stents, a key consideration in cases of contaminated wounds or concomitant infection. That said, vein grafts are not entirely immune to complications: they can develop anastomotic narrowing or late fibrosis, and graft kinking or pseudointimal hyperplasia may occur, especially if the graft is long or placed under tension. Therefore, even after open repair, lifelong surveillance is recommended. Typically, follow-up involves periodic carotid duplex ultrasounds to monitor graft or stent patency and detect any restenosis early. Intervention (angioplasty, re-stenting, or even redo surgery) can be planned if a high-grade restenosis is identified before it leads to symptoms.

The long-term outlook in this adult trauma patient is optimistic, we anticipate the vein graft will remain patent for years, as supported by prior studies of carotid reconstructions [11]. Nonetheless, restenosis can occur in any repaired artery, so our patient will require lifetime surveillance. Should significant restenosis or occlusion occur in the graft, further intervention could be needed. However, having an autologous conduit in place preserves many future options (including endovascular touch-up or redo surgery) with reduced concern for infection or device-related complications.

Our case illustrates that a staged hybrid method can achieve both immediate safety and lasting efficacy. The patient’s long-term outcome will depend on graft health and cerebral circulation status, but with prudent follow-up, we expect a favorable prognosis, including a low risk of stroke and sustained, patent carotid circulation.

## Conclusions

This case demonstrates the successful management of a traumatic CCA pseudoaneurysm in an adult using a single-stage hybrid approach, initial endovascular stenting to achieve hemorrhage control and maintain cerebral perfusion, followed by definitive open surgical reconstruction with a superficial femoral vein graft. The patient recovered without new neurologic deficits, and imaging confirmed a patent reconstruction at discharge while on dual antithrombotic therapy (aspirin + rivaroxaban). To our knowledge, this represents the first reported instance of a covered stent used as a temporary bridge to open repair in a traumatic carotid injury. This strategy underscores the evolving role of hybrid techniques in vascular trauma, offering a flexible and patient-tailored solution in anatomically or physiologically challenging scenarios.

Hybrid interventions in vascular surgery have advanced significantly over the past 50 years and have become a mainstay in the revascularization of complex vascular disease. These procedures remain integral to contemporary vascular practice and will undoubtedly continue to evolve as a cornerstone of the future of vascular surgery.
